# Effects of Cytochrome P450 2C19 and Paraoxonase 1 Polymorphisms on Antiplatelet Response to Clopidogrel Therapy in Patients with Coronary Artery Disease

**DOI:** 10.1371/journal.pone.0110188

**Published:** 2014-10-16

**Authors:** Damrus Tresukosol, Bhoom Suktitipat, Saowalak Hunnangkul, Ruttakarn Kamkaew, Saiphon Poldee, Boonrat Tassaneetrithep, Atip Likidlilid

**Affiliations:** 1 Division of Cardiology, Department of Internal Medicine, Faculty of Medicine, Siriraj Hospital, Mahidol University, Siriraj, Bangkoknoi, Bangkok, Thailand; 2 Department of Biochemistry, Faculty of Medicine, Siriraj Hospital, Mahidol University, Siriraj, Bangkoknoi, Bangkok, Thailand; 3 Integrative Computation BioScience Center (ICBS), Mahidol University, Salaya, Nakhon Prathom, Thailand; 4 Department of Health Research and Development, Faculty of Medicine, Siriraj Hospital, Mahidol University, Siriraj, Bangkoknoi, Bangkok, Thailand; University of Bologna, Italy

## Abstract

Clopidogrel is an antiplatelet prodrug that is recommended to reduce the risk of recurrent thrombosis in coronary artery disease (CAD) patients. Paraoxonase 1 (PON1) is suggested to be a rate-limiting enzyme in the conversion of 2-oxo-clopidogrel to active thiol metabolite with inconsistent results. Here, we sought to determine the associations of *CYP2C19* and *PON1* gene polymorphisms with clopidogrel response and their role in ADP-induced platelet aggregation. Clopidogrel response and platelet aggregation were determined using Multiplate aggregometer in 211 patients with established CAD who received 75 mg clopidogrel and 75–325 mg aspirin daily for at least 14 days. Polymorphisms in *CYP2C19* and *PON1* were genotyped and tested for association with clopidogrel resistance. Linkage disequilibrium (LD) and their epistatic interaction effects on ADP-induced platelet aggregation were analysed. The prevalence of clopidogrel resistance in this population was approximately 33.2% (n = 70). The frequencies of CYP2C19*2 and *3 were significantly higher in non-responder than those in responders. After adjusting for established risk factors, CYP2C19*2 and *3 alleles independently increased the risk of clopidogrel resistance with adjusted ORs 2.94 (95%CI, 1.65–5.26; p<0.001) and 11.26 (95%CI, 2.47–51.41; p = 0.002, respectively). Patients with *2 or *3 allele and combined with smoking, diabetes and increased platelet count had markedly increased risk of clopidogrel resistance. No association was observed between *PON1* Q192R and clopidogrel resistance (adjusted OR = 1.13, 95%CI, 0.70–1.82; p = 0.622). Significantly higher platelet aggregation values were found in CYP2C19*2 and *3 patients when compared with *1/*1 allele carriers (p = 1.98×10^−6^). For *PON1* Q192R genotypes, aggregation values were similar across all genotype groups (p = 0.359). There was no evidence of gene-gene interaction or LD between *CYP2C19* and *PON1* polymorphisms on ADP-induced platelet aggregation. Our findings indicated that only CYP2C19*2 and *3 alleles had an influence on clopidogrel resistance. The risk of clopidogrel resistance increased further with smoking, diabetes, and increased platelet count.

## Introduction

Acute coronary syndromes (ACS), the leading cause of sudden death worldwide, including Thailand [1], occurs as a result of platelet aggregation (thrombosis) within the human artery. Clopidogrel and aspirin are dual antiplatelet therapy that inhibit platelet function, preventing ischemic events and improving outcomes following ACS and percutaneous coronary intervention (PCI) with stent implantation [2]. Clopidogrel is a thienopyridine prodrug that requires enzymatic biotransformation into the active thiol metabolite to inhibit platelet ADP P2Y12 receptor. Aspirin (acetylsalicylic acid) is a cyclooxygenase-1 (COX-1) inhibitor, thereby preventing the production of thromboxane A_2_, which plays a prominent role in platelet aggregation. Due to the different pathways that clopidogrel and aspirin inhibit platelet aggregation, combined antiplatelet therapy provides additive benefit compared with either agent alone and is considered as a therapy of choice for preventing thrombosis in patients undergoing coronary stenting [3]. However, inter-individual variability in the response to clopidogrel is multifactorial and can be influenced by environmental, clinical, and genetic factors [4]–[6]. Many investigations have indicated that 4% to 44% of patients fail to attain platelet inhibition after clopidogrel therapy [7]–[12]. Recent studies have confirmed that *in vivo* bioactivation of clopidogrel is a two-step process which is closely linked to the cytochrome P450 (CYP) 2C19 enzyme [13]. The common genetic variants within the CYP2C19 gene, the loss-of-function hepatic CYP2C19*2 (rs4244285) and *3 (rs4986893) polymorphisms were found to be dominantly associated with a lower clopidogrel responsiveness [14]–[16] and a higher risk of adverse cardiac events such as the occurrence of stent thrombosis and recurrent myocardial infarction [17]–[19].

Recently, Bouman et al [20] reported that clopidogrel metabolism involved in two steps of bioactivation. First, clopidogrel undergoes oxidation to 2-oxo-clopidogrel by hepatic CYP450 enzyme. Then, in the second step, PON1 and PON3, the paraoxonases synthesized in the liver associated with HDL, play a crucial role in clopidogrel biotransformation to convert clopidogrel to its thiol active metabolite. Contrary to the prior observations, Bouman identified PON1 Q192R (rs662) as a single key factor for the bioactivation and clinical response of clopidogrel, and found no evidence for CYP2C19 involvement in this step of clopidogrel activation. Specifically, carriers of the QQ genotype were found to have a significantly higher risk of stent thrombosis after PCI as compared with individual with QR or RR genotype with an odds ratio (OR) of 3.3 (95% CI, 1.6–7.9; p = 0.003). However, other investigators had found no association between PON1 Q192R genotype and platelet response to clopidogrel in either Caucasian populations or populations with mixed racial background [21]–[23]. This may be due to the lower enzymatic activity of Q allele in a dose dependent manner (QQ<QR<RR) [24], [25]. Additionally, PON1 also contains the antioxidant property by breaking down biologically active oxidized phospholipids and oxidized cholesteryl esters [26], thereby preventing oxidation of HDL and LDL. Therefore, PON1 has been proposed as an atherosclerotic susceptibility gene. Many studies have reported the association between PON1 Q192R polymorphism and coronary artery disease (CAD) with mixed results. A meta-analysis of 39 studies (10,738 cases and 17,068 controls) reported a pooled OR of 1.10 (95%CI, 1.06–1.13; p<0.001) per R allele for CAD [27]. The prospective REGRESS study in 739 secondary prevention patients reported a hazard ratio (HR) of 1.71 (95%CI, 1.0–2.8; p = 0.03) per Q allele for death due to ischemic disease [28]. The GeneBank study in 1,399 sequential patients undergoing diagnostic coronary angiography reported that the Q allele was associated with an increased risk of major adverse cardiovascular events (HR, 1.48; 95%CI, 1.09–2.03; p = 0.01) [25]. This discrepancy may be due to the *PON1* allele frequency which vary greatly across human populations; a relatively high frequency of the *PON1* R192 allele is reported in Blacks, Japanese, Chinese and Thai ranging from 58% to 65% [29]–[31] as compared with Caucasians (25% to 30%) [32]. The frequency of *CYP2C19* alleles associated with poor metabolizer phenotype also showed high variability from 2–6% in Caucasians to 13–23% in Asians [33]. Since most studies were in Caucasians, there was a paucity of data in Asian populations who have different genetic background. Therefore, the aim of this study was to investigate the impact and interaction of *PON1* Q192R, CYP2C19*2 and CYP2C19*3 genotypes on clopidogrel platelet inhibition using multiple electrode platelet aggregometry (MEA) in Thai population.

## Methods

### Study population

211 patients who resided in Bangkok with aged-range from 39–94 years were recruited if they had established CAD and were on dual antiplatelet therapy with clopidogrel 75 mg and aspirin 75–325 mg daily at least 14 days prior to enrollment for secondary prevention. Subjects were excluded if they had a history of drug or alcohol abuse, bleeding disorder, current warfarin use, myelodysplastic or myeloproliferative disorders, chronic liver disease or any contraindication against aspirin or clopidogrel. Subjects were also excluded if they were pregnant, if the platelet count was less than 10^5^ cell/mm^3^ (thrombocytopenia), or if there was prior usage of glycoprotein IIb/IIIa antagonist. Questionnaires and medical records were used to collect family and medical history, smoking habit, platelet count, diabetic status, and physical activities. The study protocols were approved by Siriraj Institutional Review Board, Faculty of Medicine Siriraj Hospital, Mahidol University. Informed consent was signed by all subjects after explanation on aims and benefits of this research project.

### Platelet aggregation assays

After 14 days of taking 75 mg clopidogrel combined with 75–325 mg aspirin daily, peripheral venous blood samples were obtained from subjects in a catheterization laboratory prior to the next dose of clopidogrel and aspirin. Platelet aggregation was measured using MEA on the Multiplate analyser (Dynabyte, Munich Germany). Blood was placed in 4.5 ml plastic tubes containing hirudin with a final concentration of 25 µg/ml. The final concentration of ADP (6.5 µM) -induced platelet aggregation was assessed as previously reported [34]. Platelet aggregation measured with MEA was quantified as area under the curve (AUC = AU×min) of aggregation unit (AU). A 10 AU×min corresponds to 1 unit (U). The cut off point for this clopidogrel resistance was 50 U as previously reported [35]. All material used for platelet aggregation study was obtained from the manufacturer.

### Genotyping

Genomic DNA was isolated from whole blood by guanidine-HCl methods. Subjects were genotyped for CYP2C19*2 (681 G>A), CYP2C19*3 (636 G>A), and *PON1* Q192R (575 A>G) using PCR-RFLP as previously described [36]–[38]. Sequence specific primers were used to amplify the alleles of interest. Primers 5′ AATTACAACCAGAGCTTGGC 3′ and 5′ TATCACTTTCCATAAAAGCAAG 3′ were used to amplified the sequence of the CYP2C19*2 in exon 5 of the gene. Primers 5′ AAATTGTTTCCAATCATTTAGCT 3′ and 5′ ACTTCAGGGCTTGGTCAATA 3′ were used to amplified the sequence of the CYP2C19*3 in exon 4. Primers 5′ TATTGTTGCTGTGGGACCTGAG 3′ and 5′ CCTGAGAATCTGAGTAAATCCACT 3′ were used to amplify the sequence of the *PON1* gene containing the Q192R polymorphism in exon 6. PCR cycles for denaturation, annealing and extension were 35 cycles for all polymorphism with initial denaturation at 94°C for 5 min and final extension at 72°C for 5 min. PCR profile of CYP2C19*2 polymorphism was denatured at 94°C for 30 sec, annealing at 60°C for 30 sec and extension at 72°C for 30 sec. PCR profile for CYP2C19*3 polymorphism was denatured at 94°C for 30 sec, annealing at 58°C for 30 sec and extension at 72°C for 30 sec. For *PON1* polymorphism, denaturation was at 94°C for 1 min, annealing at 60°C for 1 min, and extension at 72°C for 30 sec. The PCR product for CYP2C19*2, CYP2C19*3 and *PON1* were 169, 271 and 238 bp, and were cut by 10 units of *SmaI*, *BamHI*, and *BspPI* restriction enzymes, respectively. Products from *SmaI* enzyme were 120 and 49 bp for G allele and 169 bp for A allele. For *BamHI*, the products were 175 and 96 bp for G allele and 271 bp for A allele and the products from *BspPI* were 175 and 63 bp for R192 allele and 238 bp for Q192 allele. The restriction site cut products were detected by 3.5% agarose gel electrophoresis.

### Statistical analyses

Variables were presented as mean ± standard deviation (SD). Chi-square goodness-of-fit test or Fisher's exact test was used to test for a possible deviation of genotype distribution from Hardy-Weinberg equilibrium (HWE) proportions. Normally distributed continuous variables were compared across two groups with the two-sided student's t-test and for genotype group comparisons with the one-way ANOVA test. The differences in allele and genotype frequencies between groups were compared using Chi-square test. A nominal p value <0.05 was considered statistically significant.

Univariable and multivariable logistic regression analyses were applied to examine whether *PON1* Q192R, CYP2C19*2 and *3 genotypes were associated with clopidogrel resistance after adjusting for age, sex, diabetes, smoking status and platelet count, assuming an additive genetic model coded as the number of mutated allele. Bonferroni's method was used for multiple testing correction considering three genetic loci tests. Statistical significant level was set at p≤0.017.

Interaction between *PON1* variants and CYP2C19*2 and *3 was performed using Cordell's test for epistatic interactions [39], using models containing two genetic markers with and without interaction term and covariates (age, sex, diabetic status, smoking status, and platelet count). Likelihood ratio test was performed with 10,000 permutations to calculate the empirical significance of the interaction term, and empirical statistically significant level was set to p<0.05. All analyses were performed using SPSS 13 (SPSS Inc. Chicago, IL, USA) and R version 2.14.2. Cordell's test was performed using *scrime* package in R [40], [41]. To determine the extent of linkage disequilibrium (LD) in our samples, standardized LD coefficient (D′) and correlation coefficient (r) were calculated for all pairs of polymorphism.

## Results

### Baseline characteristics of study participants

Based on the result from platelet function test using MEA, the CAD patients were categorized into responders and non-responders to clopidogrel. Among 211 patients included in this study, 70 patients (33.2%) were classified as non-responders and 141 patients (66.8%) as responders. There was no significance between the two groups regarding differences in age, BMI, sex, number of vessel diseases, underlying diseases (cardiomyopathy, hypertension, dyslipidemia, stroke, renal impairment, and peripheral disorder), and concurrent medications (p>0.05). However, clopidogrel non-responders had a significantly higher proportion of diabetes (p = 0.002), smokers (p = 0.043), and higher platelet counts (p = 0.033) as shown in Table 1.

**Table 1:** Baseline characteristics of study participants.

	Total	Non-responders	Responders	
Parameters	(n = 211)	(n = 70)	(n = 141)	p-value*
**Age**	66.25±11.15	64.47±10.51	67.13±11.39	0.102
**BMI (kg/m^2^)**	25.54±4.08	25.59±4.13	25.57±4.08	0.970
**Female (%)**	68 (32.3)	21 (30.0)	47 (33.3)	0.626
**Type of CAD**				
** - Single vessel disease (%)**	51 (24.1)	19 (27.1)	32 (22.7)	0.477
** - Multi vessel disease (%)**	143 (67.8)	46 (65.7)	97 (68.8)	0.757
** - Others (%)**	17 (8.1)	5 (7.1)	12 (8.5)	0.731
**Cardiomyophaty (%)**	7 (3.3)	3 (4.3)	4 (2.8)	0.580
**Diabetes (%)**	97 (46.0)	43 (61.4)	54 (38.3)	0.002*
**Hypertension (%)**	184 (87.8)	61 (87.1)	123 (87.2)	0.985
**Dyslipidemia (%)**	149 (70.6)	52 (74.3)	97 (68.8)	0.410
**Stroke (%)**	14 (6.6)	6 (8.6)	8 (5.7)	0.426
**Renal impairment (%)**	23 (10.9)	6 (8.6)	17 (12.1)	0.444
**Peripheral arterial disorder (%)**	13 (6.2)	4 (5.7)	9 (6.4)	0.849
**Smoking (%)**	85 (40.3)	35 (50.0)	50 (35.5)	0.043*
**Medication**				
**- Proton pump inhibitors (%)**	83 (39.3)	30 (42.9)	53 (37.6)	0.461
**- Calcium channel blockers (%)**	67 (31.8)	22 (31.4)	45 (31.9)	0.943
**- Statin (%)**	183 (86.7)	65 (92.9)	118 (83.7)	0.065
**Platelet count (**×10^5^/**mm^3^)**	2.55±0.76	2.73±0.84	2.47±0.72	0.033*
**ADP platelet aggregation (U)**	43.98±26.19	73.33±18.26	28.95±10.42	<0.001

* Variable is significant difference between responders and non-responders at p-value<0.05.

### Distribution and allele frequencies of CYP2C19*2, *3 and *PON1* Q192R genotypes

The distribution of CYP2C19*2, *3 and *PON1* Q192R genotypes in the clopidogrel responsive and non-responsive groups were summarized in Table 2, which indicates consistency with the Hardy-Weinberg equilibrium (p>0.05). There was no homozygous CYP2C19*3 genotype detected in the study population, which is consistent with its very rare frequency in Caucasians, Africans, Americans, Japanese and Koreans. Moreover, the high frequency of *PON1* R192 in this study was consistent with the other reports in Asian populations. The frequencies of both *2/*2 and *2/*3 genotypes (17.10, 10.00 vs 2.10, 0.70%) and *2 and *3 alleles (39.29, 7.14 vs 20.92, 1.42%) were significantly higher in clopidogrel non-responders than those in responders (p = 1.6×10^−4^, p = 2.1×10^−3^ and p = 6.5×10^−5^, p = 3.6×10^−4^, respectively). Similarly, the frequencies of CYP2C19*1 genotype and allele (34.30, 53.57 vs 58.20, 77.66%) were significantly lower in non-responders than those in responders (p = 1.1×10^−3^ and 4.0×10^−7^, respectively). There were no significant differences of *PON1* Q192R genotypes and alleles between the two groups (p>0.05).

**Table 2:** Distribution of CYP2C19*2, *3 and PON1 genotypes in clopidogrel responders and non-responders.

CYP2C19*2 (rs4244285)				
	Non-responders	Responders	Total	
Genotype	(n = 70)	(n = 141)	(n = 211)	p-value*
GG (*1/*1)	27 (38.6%)	85 (60.3%)	112 (53.1%)	2.9×10^−3^ †
GA (*1/*2)	31 (44.3%)	53 (37.6%)	84 (35.8%)	0.349
AA (*2/*2)	12 (17.1%)	3 (2.1%)	15 (7.1%)	1.6×10^−4^ †
**HWE p-value** ‡	0.549	0.106	0.889	-
**Allele frequency**				
Allele*2 (95%CI)	0.39 (0.32–0.48)	0.21 (0.16–0.26)	0.27 (0.23–0.31)	6.3×10^−5^ †

CYP2C19*3 (rs4986893)				
	Non-responders	Responders	Total	
Genotype	(n = 70)	(n = 141)	(n = 211)	p-value*
GG (*1/*1)	60 (85.7%)	137 (97.2%)	197 (93.4%)	2.9×10^−3^ †
GA (*1/*3)	10 (14.3%)	4 (2.8%)	14 (6.6%)	2.9×10^−3^ †
AA (*3/*3)	0 (0)	0 (0)	0	-
**HWE p-value** ‡	0.520	0.864	0.618	-
**Allele frequency**				
Allele*3 (95%CI)	0.07 (0.03–0.11)	0.01 (0.0003–0.03)	0.03 (0.02–0.05)	3.4×10^−3^ †

CYP2C19*2 and *3 Combination				
	Non-responders	Responders	Total	
Genotype	(n = 70)	(n = 141)	(n = 211)	p-value*
*1/*1	24 (34.3%)	82 (58.2%)	106 (50.2%)	1.1×10^−3^ †
*1/*2	24 (34.3%)	52 (36.9%)	76 (36.0%)	0.712
*1/*3	3 (4.3%)	3 (2.1%)	6 (2.9%)	0.401
*2/*2	12 (17.1%)	3 (2.1%)	15 (7.1%)	1.6×10^−4^ †
*2/*3	7 (10.0%)	1 (0.7%)	8 (1.8%)	2.1×10^−3^ †
*3/*3	0 (0)	0 (0)	0	-
**HWE p-value** ‡	0.127	0.451	0.083	-
**Allele frequency**				
Allele*1 (95%CI)	0.54 (0.45–0.62)	0.78 (0.72–0.83)	0.70 (0.65–0.74)	4.0×10^−7^ †
Allele*2 (95%CI)	0.39 (0.31–0.47)	0.21 (0.16–0.26)	0.27 (0.23–0.31)	6.5×10^−5^ †
Allele*3 (95%CI)	0.07 (0.04–0.10)	0.01 (0.0003–0.02)	0.03 (0.02–0.05)	3.6×10^−4^ †

PON1 (Q192R; rs662)				
	Non-responders	Responders	Total	
Genotype	(n = 70)	(n = 141)	(n = 211)	p-value*
**GG (RR)**	34 (48.6%)	71 (50.4%)	105 (49.8%)	0.807
**AG (QR)**	29 (41.4%)	55 (39.0%)	84 (39.8%)	0.735
**AA (QQ)**	7 (10.0%)	15 (10.6%)	22 (10.4%)	0.886
**HWE p-value** ‡	0.824	0.381	0.399	0.886
**Allele frequency**				
**Allele Q (95%CI)**	0.31 (0.24–0.38)	0.30 (0.25–0.35)	0.30 (0.26–0.34)	0.904

* Comparison of genotype and allele frequencies between non-responders and responders.

† Statistically significant difference at p<0.05.

‡ p-value of Hardy-Weinberg equilibrium.

### Association of *CYP2C19* and *PON1* Q192R gene polymorphisms and clopidogrel responsiveness

The results of a simple logistic regression model demonstrated that having one copy of CYP2C19*3 was significantly associated with a 5.71 fold higher risk of clopidogrel resistance (95% CI, 1.72–18.93; p = 0.004) as compared with wild type *CYP2C19* (*1/*1). Although one copy of CYP2C19*2 was not significantly associated with clopidogrel resistance (p = 0.053), two copy of CYP2C19 (*2/*2) was associated with 12.59 times higher risk of clopidogrel resistance (95%CI, 3.31–47.96; p<0.001). The combined effect of CYP2C19*2 and *3 estimated that both *2/*2 and *2/*3 genotypes significantly increased the risk of clopidogrel resistances with an unadjusted OR of 13.67 (95%CI, 3.56–52.43; p<0.001) and 23.92 (95%CI, 2.80–204.11; p = 0.004), respectively. After adjusting for the co-dominant effects of *2 and *3 alleles, comparing to *1 allele, *2 was associated with 2.63 times higher risk of clopidogrel resistance (95%CI, 1.62–4.27; p<0.001), and *3 was associated with 6.18 times higher risk of clopidogrel resistance (95%CI, 1.80–21.17; p = 0.004). In contrast, the *PON1* Q192R, both genotypes (QQ/QR) and Q allele, did not significantly associate with clopidogrel resistance (p>0.05).

From multivariable logistic regression analysis, assuming a co-dominant allele effect, having one copy of CYP2C19*2 (*1/*2) was associated with 2.30 times higher risk than *1/*1 (95%CI, 1.14–4.66); p<0.021), after adjusted for age, sex, and all variables that differed between responders and non-responders (from Table 1). Similarly, one copy of CYP2C19*3 (*1/*3) was associated with 10.59 times higher risk of clopidogrel resistance compared with *1/*1 (95%CI, 2.39–46.85; p = 0.002). Two copy of CYP2C19*2 (*2/*2) was associated with 13.23 times higher risk of clopidogrel resistance compared with *1/*1 (95%CI, 2.87–60.88; p = 0.001).

The combined effects of CYP2C19*2 and *3, after controlling for additional covariates, compared with *1/*1, CYP2C19*2/*3 was associated with 84.06 times higher risk of clopidogrel resistance (95%CI, 6.89–1026.24; p = 0.001); homozygous CYP2C19*2 (*2/*2) was associated with 13.09 times higher risk of clopidogrel resistance (95%CI, 2.83–60.57; p = 0.001).

For allelic association, after adjusting for the co-dominant effect of *3 allele, *2 allele carrier was associated with 2.94 times higher risk of clopidogrel resistance compared with *1 allele (95%CI, 1.65–5.26; p<0.001). After adjusting for the effects of *2 allele, *3 allele was associated with 11.26 times higher risk of clopidogrel resistance compared with *1 (95%CI, 2.47–51.40; p = 0.002). In contrast, PON1 QR and QQ genotypes and Q allele showed no association with clopidogrel resistance compared with either RR genotype or R allele as references (Table 3).

**Table 3:** Association between CYP2C19*2, *3, PON1 Q192R and clopidogrel resistance.

CYP2C19*2 (rs4244285)				
Genotype	Crude OR (95%CI)	p-value	Adjusted OR (95%CI)†	p-value
GG (*1/*1)	1	-	1	-
GA (*1/*2)	1.84 (0.99–3.42)	0.053	2.30 (1.14–4.66)	0.021
AA (*2/*2)	12.59 (3.31–47.96)	<0.001*	13.23 (2.87–60.88)	0.001*
Allele A (*2)	2.57 (1.59–4.14)	<0.001*	2.86 (1.63–5.03)	<0.001*

CYP2C19*3 (rs4986893)				
Genotype	Crude OR (95%CI)	p-value	Adjusted OR (95%CI)†	p-value
GG (*1/*1)	1	-	1	-
GA (*1/*3)	5.71 (1.72–18.93)	0.004*	10.59 (2.39–46.85)	0.002*
AA (*3/*3)	-	-	-	-
Allele A (*3)	5.71 (1.72–18.93)	0.004*	10.59 (2.39–46.85)	0.002*

CYP2C19*2 and *3 combination				
Genotype	Crude OR (95%CI)	p-value	Adjusted OR (95%CI)†	p-value
*1/*1	1	-	1	-
*1/*2	1.58 (0.81–3.06)	0.179	2.01 (0.95–4.29)	0.070
*1/*3	3.42 (0.65–18.04)	0.148	3.70 (0.46–30.01)	0.221
*2/*2	13.67 (3.56–52.43)	<0.001*	13.09 (2.83–60.57)	0.001*
*2/*3	23.92 (2.80–204.11)	0.004*	84.06 (6.89–1026.24)	0.001*
Allele *2 ‡	2.63 (1.62–4.27)	<0.001*	2.94 (1.65–5.26)	<0.001*
Allele *3 ‡	6.18 (1.80–21.17)	0.004*	11.26 (2.47–51.40)	0.002 *

*PON1* (Q192R; rs662)				
Genotype	Crude OR (95%CI)	p-value	Adjusted OR (95%CI)†	p-value
GG (RR)	1	-	1	-
AG (QR)	1.10 (0.60–2.02)	0.756	1.36 (0.68–2.72)	0.381
AA (QQ)	0.98 (0.36–2.61)	0.959	1.05 (0.35–3.16)	0.925
Allele A (Q)	1.03 (0.67–1.57)	0.907	1.13 (0.70–1.82)	0.622

* Risk is statistical significant when compared to the reference genotype at p-value<0.017.

† Adjusted for diabetes, age, sex, history of smoking and platelet count.

‡ Adjusted for concurrent *2 or *3 allele and covariates (diabetes, age, sex, history of smoking, and platelet count).

The estimated effects of *1, *2, and *3 genotypes, combined with smoking, diabetes status, and increase in platelet count using *1/*1 as a reference genotype, markedly increase the risk of clopidogrel resistance in linear trend as summarized in Table 4.

**Table 4:** Estimated risk of clopidogrel resistance in patients with at least one of the following risk factors: CYP2C19*2, CYP2C19*3, smoking, diabetes mellitus, increase in platelet count adjusted for age and sex.

Risk Factors	Adjusted OR*	95% CI	p-value†
*1/*1	1	-	-
*1/*1+Smoking	3.52	1.53–8.09	0.003
*1/*1+DM	3.33	1.62–6.85	0.001
*1/*1+Platelet	1.05	1.00–1.10	0.034
*1/*1+DM+Smoking	7.78	2.68–22.53	<0.001
*1/*1+DM+Platelet	3.26	1.65–6.45	0.001
*1/*1+DM+Smoking+Platelet	8.12	2.82–23.73	<0.001
*1/*2	2.94	1.65–5.26	<0.001
*2/*2	8.78	3.00–25.71	<0.001
*1/*3	11.26	2.47–51.41	0.002
*2/*3	33.15	7.01–156.72	<0.001
*1/*2+Smoking	7.43	2.76–20.05	<0.001
*2/*2+Smoking	22.03	5.44–89.17	<0.001
*1/*3+Smoking	28.06	4.84–162.83	<0.001
*2/*3+Smoking	83.16	12.51–552.97	<0.001
*1/*2+DM	9.19	3.59–23.52	<0.001
*2/*2+DM	27.23	6.90–107.37	<0.001
*1/*3+DM	34.68	6.75–178.06	<0.001
*2/*3+DM	102.77	17.16–615.29	<0.001
*1/*2+Platelet count	3.12	1.82–5.34	<0.001
*2/*2+Platelet count	9.23	3.15–27.03	<0.001
*1/*3+Platelet count	11.76	2.81–49.29	<0.001
*2/*3+Platelet count	34.86	7.30–166.45	<0.001
*1/*2+Smoking+DM	23.05	6.36–83.56	<0.001
*2/*2+Smoking+DM	68.3	12.96–360.05	<0.001
*1/*3+Smoking+DM	86.99	12.30–615.28	<0.001
*2/*3+Smoking+DM	257.79	31.21–2129.62	<0.001
*1/*2+Smoking+DM+Platelet	24.23	6.67–87.95	<0.001
*2/*2+Smoking+DM+Platelet	71.82	13.62–378.77	<0.001
*1/*3+Smoking+DM+Platelet	91.47	12.82–652.52	<0.001
*2/*3+Smoking+DM+Platelet	271.07	32.56–2256.56	<0.001

*Estimated OR for each risk factor category compared to men with no CYP2C19 mutation (wild type), with average age (66.25 year-old), average platelet count (255,900 platelets/mm^3^), who do not smoke and do not have diabetes. Platelet variable is calculated per ×1,000 platelet increased. Log odds for clopidogrel resistance were calculated using multivariate logistic regression as a function of CYP2C19*2+CYP2C19*3+Smoking+Diabetic Status+increased Platelet Count.

† Risk is statistically significant when compared to the reference genotype at p-value<0.05.

### CYP2C19*2, *3 and PON1 Q192R genotypes and platelet aggregation

The ADP-induced platelet aggregation values across CYP2C19*2 and *3 genotypes were shown in Table 5. For CYP2C19*2 genotypes, ADP-induced platelet aggregation significantly differed across genotype groups (p = 2.98×10^−5^). In the patients who were carriers of at least one *2 allele (*1/*2 or *2/*2), ADP induced-platelet aggregation was also significantly different when compared with *1/*1 genotype (p = 0.004). For CYP2C19*3 genotypes, the ADP-induced platelet aggregation did not differ across genotype groups (p = 0.069). However, when combining CYP2C19*2 and *3, the ADP-induced platelet aggregations across genotypes were significantly different (p = 1.98×10^−6^). For *PON1* Q192R genotypes, the ADP-induced platelet aggregation did not differ across genotype groups (p = 0.359).

**Table 5:** ADP induced platelet aggregation level by CYP2C19*2, *3 and PON1 Q192R polymorphisms in clopidogrel treated patients with coronary artery disease.

	Genotypes	n	Platelet Aggregation Level
***CYP2C19*** *2	GG (*1/*1)	112	39.16±23.28
(rs4244285; 681G>A)	GA (*1/*2)	84	45.20±23.62
	AA (*2/*2)	15	73.07±39.77
	p-value		2.98×10^−5^
***CYP2C19*** *3	GG (*1/*1)	197	43.10±26.36
(rs4986893; 636G>A)	GA (*1/*3)	14	56.29±20.69
	AA (*3/*3)	0	-
	p-value		0.069
***CYP2C19*** *2	*1/*1	106	38.90±23.49
**& ** ***CYP2C19*** *3	*1/*2	76	43.36±23.28
	*1/*3	6	47.67±19.14
	*2/*3	8	62.75±20.54
	*2/*2	15	73.07±39.77
	p-value		1.98×10^−6^
***PON1***	GG (RR)	105	42.14±25.43
(rs662; 575A>G)	AG (QR)	84	45.77±27.30
	AA (QQ)	22	45.86±26.08
	p-value		0.359

p-values assuming additive genetic model represent the association between genotype and ADP-induced platelet aggregation (U) at p-value<0.05.

### Interaction between *PON1* Q192R polymorphisms and ADP-induced platelet aggregation level after stratification by CYP2C19*2 and CYP2C19*3

**Figure 1:**
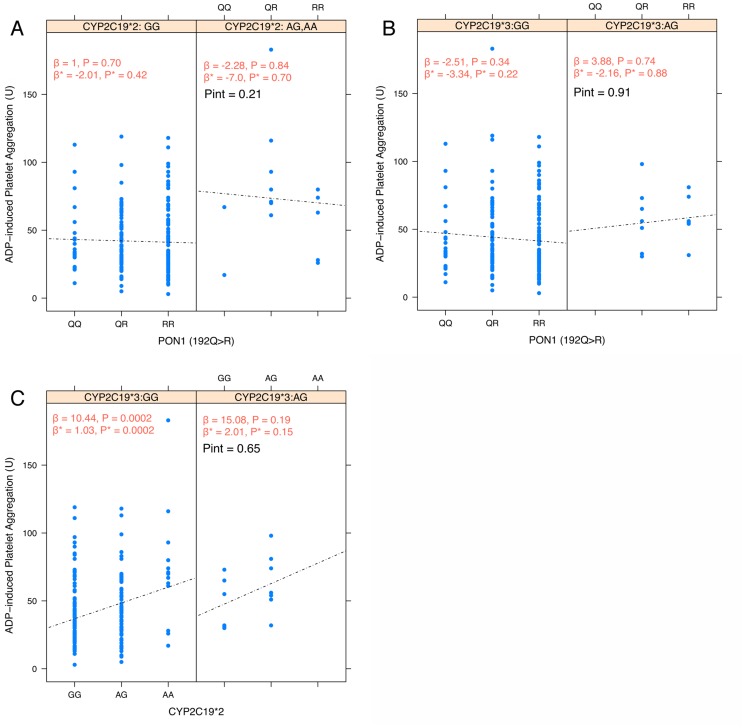
Interaction among polymorphisms in CYP2C19*2, *3 and PON1 Q192R and the effects on ADP-induced platelet aggregation. A) Effects of PON1 Q192R polymorphism on platelet aggregation stratified by CYP2C19*2 genotype; B) Effects of *PON1* Q192R polymorphism on platelet aggregation stratified by CYP2C19*3 genotype; C) Effects of CYP2C19*2 on platelet aggregation stratified by CYP2C19*3 genotype

Since both CYP2C19 and PON1 involve in activation of clopidogrel prodrug as suggested by Bouman et al. [20], the interaction effects between CYP2C19 (*2, *3) and PON1 (Q192R) on ADP-induced platelet aggregation were investigated. After stratification by CYP2C19*2 (Figure 1A) and *3 genotypes (Figure 1B), the effects of PON1 (Q192R) polymorphism on ADP-induced platelet aggregation were not modified by neither CYP2C19*2 nor CYP2C19*3 allele. Cordell's test for epistatic interaction showed no statistically significant interaction between CYP2C19*2 or *3 with PON1 Q192R polymorphisms (p_int_ = 0.21 and 0.91, respectively). Similarly, CYP2C19*3 did not modify the effects of CYP2C19*2 on ADP-induced platelet aggregation (p_int_ = 0.65, Figure 1C). To examine the extent of linkage disequilibrium (LD) in these study samples, standardized LD coefficient (D′) and correlation coefficient (r) were calculated for all pairs of polymorphisms. Table 6 shows the LD matrix generated using D′ and r. No evidence of LD was observed among these three polymorphisms (D′ and r<0.5).

**Table 6:** Standardized linkage disequilibrium coefficient (D′) and correlation coefficient (r) among three polymorphisms in CYP2C19 and PON1.

D′ r	*PON1* Q192R	CYP2C19*3	CYP2C19*2
***PON1*** ** Q192R**	-	−0.0276	0.0089
***CYP2C19*** *3	0.2258	-	0.0223
***CYP2C19*** *2	0.0097	0.0731	-

*D′* values are shown in the lower triangle, and *r* values are shown in the upper triangle.

## Discussion

Bouman et al's study [20] is the first report to identify that *PON1* Q192R is a major determinant of clopidogrel efficacy using in vitro metabolomic profiling techniques. The PON1 activity was significantly reduced in subjects with homozygous wild type allele (*PON1* QQ192) compared with carriers of the mutant allele. In addition, in a group of patients with stent thrombosis and matched controls without stent thrombosis, *PON1* QQ192 was associated with decreased platelet inhibition by clopidogrel and decreased plasma active thiol metabolite after given a 600 mg clopidogrel loading dose. In addition, *PON1* QQ192 was also associated with an OR of 3.3 for the occurrence of stent thrombosis as compared with QR192 or RR192 genotypes. Later, however, other studies could not document the influence of *PON1* Q192R genotype on clopidogrel antiplatelet aggregation since the publication of the study by Bouman et al [21]–[23]. In this study, we evaluated the effects of *CYP2C19* and *PON1* genetic polymorphisms on clopidogrel antiplatelet function in Thai population. Similar to the findings from other investigators in African-American and Caucasian populations [21], [22], our results have shown that only CYP2C19*2 and *3 genotypes, but not the *PON1* Q192R genotypes, modified the effect of clopidogrel. The mean aggregation values increased by a strong genetic effect across *CYP2C19* genotype groups in individuals treated with clopidogrel. Also, only CYP2C19*2 and *3 genotypes but not the *PON1* Q192R genotypes were found to be associated with a higher risk of clopidogrel resistance in CAD patients during treatment with clopidogrel.

The present study was strengthened by testing the influence of these SNPs on platelet aggregation in parallel as measured by MEA assay. Only CYP2C19*2 and *3 polymorphisms have been demonstrated to be a strong determinant of reduced active clopidogrel metabolite formation corresponding to the studies in Caucasians [14], [17], [42]–[52]. Nevertheless, the influence of PON1 on the level of platelet aggregation had a trend towards higher values in QR192 and QQ192 patients (Table 5). This suggested that *PON1* polymorphism may be associated with small differences in platelet inhibition as suggested by the finding of Bouman et al [20]. The small effects of *PON1* Q192R could explain why several reports were unable to confirm this association between *PON1* polymorphism and platelet aggregation in patients who were treated with clopidogrel [21]–[23],[53]–[57]. Concerning the clinical outcome of patients treated with clopidogrel, our results reported here are in agreement with a number of prior studies and confirm the pivotal role of CYP2C19*2 and *3 as genetic markers for platelet aggregation and clopidogrel response. This present study also demonstrated no association and linkage disequilibrium between *CYP2C19* and *PON1* polymorphisms, which supports the evidence that *CYP2C19* locus, located on chromosome 10, was the only locus which was significantly associated with clopidogrel treatment efficacy in a genome-wide association study (GWAS) [42]. The GWAS did not find evidence for association between SNPs located on or near the *PON1* gene on chromosome 7 and variation in platelet inhibition by clopidogrel [42]. In addition, in a meta-analysis investigating the effect of *CYP2C19* alleles on recurrent stenosis in patients receiving clopidogrel after coronary stenting, the presence of one reduced-function allele was associated with a HR of 2.67, and the presence of two reduced-function alleles was associated with a HR of 3.97 for the recurrence of thrombosis [46]. This study also confirms that the presence of one reduced-function allele of *CYP2C19* was associated with adjusted ORs of 2.94 and 11.26 for *2 and *3, respectively. The presence of two reduced-function alleles was associated with adjusted ORs of 13.09 and 84.06 for *2/*2 and *2/*3, respectively. These findings support the clinical importance of the reduced-function *CYP2C19* polymorphism and clopidogrel resistance on recurrent ischemic events and restenosis after coronary stenting.

In this study, smoking status, diabetes mellitus, and increase in platelet count were shown to be the three major contributing factors that could promote the development of platelet aggregation in CAD patients (Table 4). These conditions have been known to be associated with high oxidative stress, suggesting a possible link between high oxidative stress and response to clopidogrel treatment. This study suggested that not only genetic polymorphisms but also oxidative stress can enhance platelet aggregation to clopidogrel responsiveness in CAD patients.

Limitations of the study include a relatively small sample size, which might contribute to the inability to detect weaker effects of PON1 on clopidogrel response, as compared with the stronger effects of CYP2C19. Although plasma levels of the active metabolite of clopidogrel and PON1 enzyme activity were not measured to confirm the lower level of enzyme activity associated with Q allele, these parameters could be used to indirectly assess the platelet function test as measured by ADP-induced platelet aggregation. Finally, platelet function testing was done with only one single device (Multiplate anslyser), using ADP-induced platelet aggregation, therefore, we could not exclude the possibility that other mechanisms might also explain the clopidogrel resistance as measured by the Multiplate analyser.

## Conclusions

This study confirms the impact of CYP2C19*2 and *3 polymorphisms on antiplatelet effects of clopidogrel in Thai population similar to the results found in Caucasian populations with different genetic background. *PON1* Q192R appeared to have a little modification of efficacy and safety of clopidogrel in CAD patients. A larger study may be needed to confirm the association of the *PON1* Q192 allele with adverse ischemic events in patients receiving clopidogrel treatment. Our results are only relevant to clopidogrel-treated patients; however, knowing the genotypes of *CYP2C19* should aid in selection of antiplatelet therapy. In the future, pharmacogenetic studies may be needed to introduce newer antiplatelet drugs that do not require *CYP2C19* activation and may reduce the overall impact of clopidogrel resistance in patients with CAD.
